# Long-term Results After Transcatheter Aortic Valve Implantation: What do we Know Today?

**DOI:** 10.2174/1573403X09666131202124227

**Published:** 2013-11

**Authors:** Y. Elhmidi, S. Bleiziffer, N. Piazza, B. Voss, M. Krane, M-A. Deutsch, R. Lange

**Affiliations:** Department of Cardiovascular Surgery, German Heart Centre Munich, Munich, Germany

**Keywords:** Aortic valve, TAVI, paravalvular leakage, aortic stenosis, SAVR.

## Abstract

Transcatheter aortic valve implantation (TAVI) is evolving rapidly as a therapeutic option in patients deemed to be at high risk for surgical aortic valve replacement. Early outcome and survival of controlled feasibility trials and single- center experience with TAVI have been previously reported. Valve performance and hemodynamics seem to improve significantly after TAVI. Long-term outcome up to 3 years have been demonstrated in recent studies. Admittedly, the results are encouraging with a survival rate at 2 and 3 years ranging from 62 to 74% and from 56 to 61% respectively. The improvement in hemodynamical and clinical status sustained beyond the 3 years follows up. However, paravalvular leakage after TAVI remains an important issue in this rapidely evolving field.

## INTRODUCTION

Aortic stenosis (AS) is the most common valvular disease in the world. The surgical therapy is currently the gold standard for the treatment of the calcified stenotic valve [1]. With medical therapy only, the prognosis is very poor with a 3-year survival rate of <30% [1]. However, more than 30% of all patients aged > 70 years presented with severe aortic stenosis are deemed to be at high risk for surgical aortic valve replacement (SAVR) [2]. Patients undergoing only medical therapy or balloon valvuloplasty, have a pour prognosis with heart failure and higher mortality [3]. 

Transcatheter aortic valve implantation is a novel and rapidly evolving technique. It was first introduced in 2002 and is currently available as an alternative to conventional AVR for patients with severe symptomatic AS who are deemed to be at high risk for open heart surgery. Several studies showed the feasibility and safety of TAVI in those patients with high STS and EuroSCORES (scores for predicted operative mortality) [4, 5]. Patients following TAVI demonstrated hemodynamics and symptoms relief [6, 7]. Moreover, an immediate left ventricular improvement has been shown as well [8-11]. Early outcome and survival data have been previously reported in small controlled feasibility trials and single-center experience reports [6, 12]. Currently, first reports about the outcomes after TAVI including patients beyond a two-year follow-up period are available.

This review summarizes the most recent data published concerning the long-term results of patients undergoing TAVI including mortality, durability and hemodynamic performance up to 3 years. 

## EARLY MORTALITY AND LONG-TERM SURVIVAL

The most recent TAVI studies showed improved 30-day and 1-year survival with growing experience. The first transfemoral and transapical TAVI experiences demonstrated a higher 30-day mortality ranging from 12 to 15% and in some series up to 20% [13, 14]. Most centres demonstrated a learning curve with better results and less intra- and postprocedural complications over time [15, 16]. Devices techniques, MSCT imaging, risk calculations, patient’s selection and the introduction of a multidisciplinary team are factors which lead to an increasing implantation success [17]. 

The published data for long-term survival are shown in Table **1**. There are very few data demonstrating the long-term survival in patients following TAVI. Most centres began with a TAVI program in 2009. Walther *et al*. presented recently the results of TAVI at 3-year follow up. A total of 299 patients underwent transapical aortic valve implantation from February 2006 until January 2010 using the Edwards SAPIEN transcatheter prosthesis. In their study, the 30-day survival was 91%, 73% at 1 year, 68% at 2 years, and 58% at 3 years [18]. Gurvitch *et al*. presented the long-term results of transfemoral and transapical Edwards Sapien implantation in one single-center in 70 patients during a mean follow up of 3.7 years. Similar to the previous study, the 1-year, 2-year and 3-year survival rates were 81%, 74% and 61%, respectively [19]. Congruently to these results, Ussia *et al*. presented the long-term outcomes of a large Italian registry undergoing TAVI with the Medtronic CoreValve device. The survival rate at 1-year, 2-year and 3-year were 76.4%, 69.7% and 56.2% respectively [20]. 

Bleiziffer *et al.* presented recently the 2-year results in 227 patients following transfemoral and transapical TAVI. The Medtronic CoreValve prosthesis was implanted in 174 patients and the Sapien Edwards prosthesis in 53 patients. The early and long-term mortality rates were not different from previous reports. The survival rates at 30-day, 1-year and 2-year were 88.5%, 74.5% and 64.4% respectively [21]. According to their findings the majority of deaths occured within the first 6 months after TAVI. 

A recent multicenter single-arm study with symptomatic patients with severe aortic stenosis undergoing TAVI demonstrates a 30-day all-cause mortality of 15.2% and a 2-year survival of 61.9% [22]. The study evaluated the durability of both safety and efficacy of TAVI using the Medtronic CoreValve prosthesis. In summary, death after TAVI occurred with an incidence density of 10.0% at 1 year and 1/3 of deaths were observed within the first 30 days after the procedure [23]. 

## DEVICE PERFORMANCE AND DURABILITY

The device performance and durability is still subject of investigation. The published TAVI studies presented excellent postoperative device function, however, long-term results of device durability are still lacking. Concerns about tissue deterioration, endocarditis and tissue injury through crimping and after balloon dilatation, frame fracture and valve migration have been largely discussed and may potentially impact long-term valve function. 

The excellent device performance for transcatheter aortic valves is inasmuch tempered by the increased risk of paravalvular leakage, caused by the native valve cusp calcifications which remain in situ. The incidence of moderate paravalvular regurgitation is ranging from 4% to 40% [24, 25]. In high risk patients with severe comorbidities, a moderate or high grade paravalvular leak is an additional risk for prolonged intubation, hospital stay, renal insufficiency and in some cases a subsequent SAVR was necessary. Indeed, most paravalvular leaks are mild to moderate depending on the implanted valves [12, 23, 26]. More importantly, patients who undergo SAVR after TAVI for severe paravalvular leak are at high risk for in-hospital morbidity and mortality [12]. The rates of moderate paravalvular leakage are shown in Table **2** and range from 4 to 15%. Usually, the initial AR after TAVI stays unchanged and no progression to severe AR is described over time [27, 28]. The recently published study from Unbehaun *et al*. [29] described the incidence and prognostic value of paravalvular leakage after TAVI. According to their findings, the cumulative survival rate was not dependent on post-procedural AR. The PARTNER Trial however raised concern that even mild aortic regurgitation was associated with late mortality after TAVI. Results after 2 years showed that mild aortic regurgitation was associated with increased long-term mortality [29].

All previous studies documented no evidence of structural valve detoriation or significant changes of the hemydynamic status of the prostheses [20-22] (Table **3**). Leaflet deterioration after TAVI has been published in only one single case report. Ong *et al*. demonstrated a remarkable degeneration of a CoreValve prosthesis five years after implantation [30]. Such cases are rare; however, this may become an issue if TAVI is applied in younger patients.

In all studies, the incidence of endocarditis was very low. Walther *et al*. reported only one case of endocarditis (0.4%) [18]. The SOURCE registry [6] reported an incidence of only 1%. Gurvitch *et al.* observed in only 1.4% of all patients a valve endocarditis. However, late follow-up data in these patients remain relatively sparse [19]. 

**Fig. (1):**
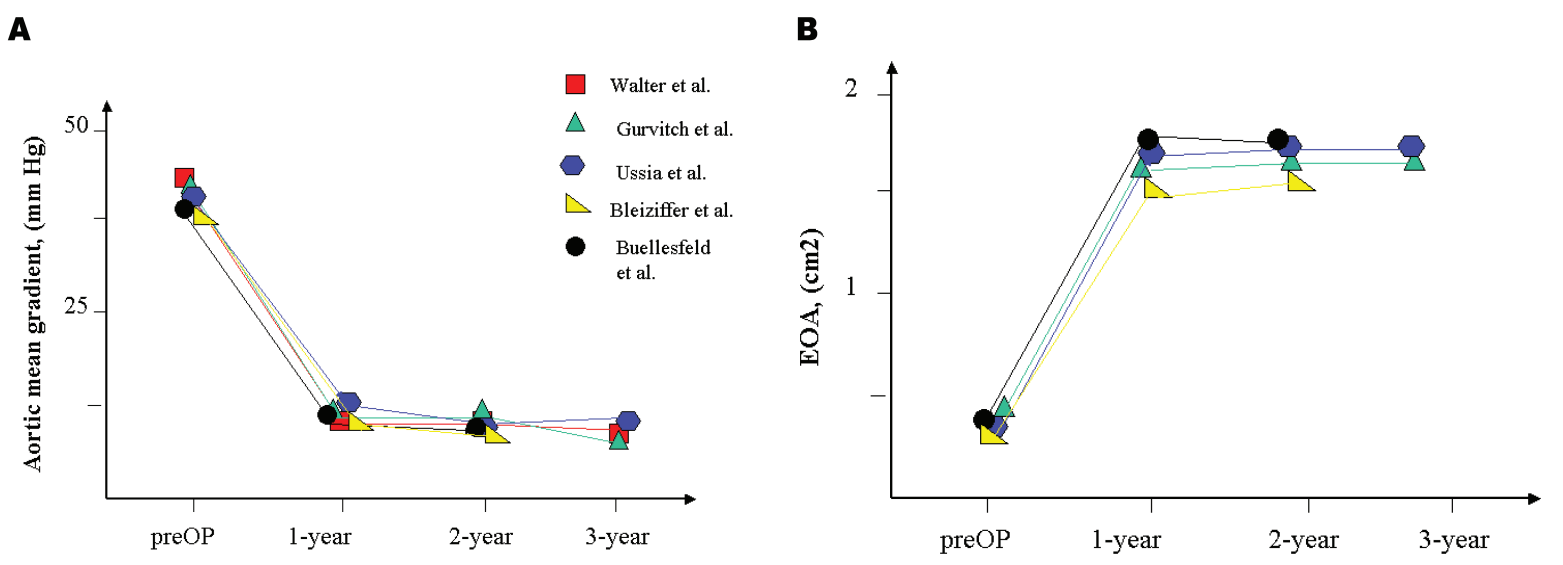
The hemodynamical changes in (A) Aortic mean gradient and (B) Effective orifice area (EOA) in all studies.

The hemodynamics and valve efficacy were assessed by echocardiographic controls up to 3 years follow up (Fig. **1**). All studies presented the effective orifice area (EOA) and transvalvular aortic mean gradients. In the study of Gurvitch *et al*., echocardiographic follow-up data were available in 37 patients. The mean aortic gradient decreased significantly from 45 mmHg to 10 mmHg after TAVI (p<0.01) with a subsequent increase to 12 mmHg after 3 years. The calculated aortic valve area increased significant from 0.6 ± 0.2 cm^2^ at baseline to 1.7 ± 0.4 cm^2^ after TAVI with a subsequent attrition at 12 months to 1.5 ± 0.3 cm^2 ^and to 1.4 ± 0.3 cm^2^ after 3 years [29]. In 89 patients who survived the 3 years follow up, Ussia *et al*. showed a significant decrease in the aortic mean gradient from 52 mmHg to 10.3 mmHg after 1 year and a gradient of 10.3 mmHg after 3 years follow up [20]. The calculated aortic valve area increased significantly from 0.6 cm^2^ at baseline to 1.7 cm^2 ^after TAVI and remained unchanged after 3 years (1.7 cm^2^). The study of Bleiziffer *et al.* confirmed all previous findings. The mean aortic gradient decreased significantly from 48 mmHg at baseline to 12 mmHg at discharge with no changes over 2 years follow up. The calculated aortic valve area increased from 0.67 cm^2 ^at baseline to 1.55 cm^2^ at discharge and remained unchanged 2 years after TAVI [21]. 

## NEUROLOGICAL EVENTS IN FOLLOW UP

The major neurological adverse outcomes are illustrated in Table **3**. In the PARTNER Trial, the neurologic events were 2-fold higher in TAVI patients than in SAVR (5.5 vs 2.4% at 30-days, p=0.04, respectively). Moreover, 50% of all strokes occurred during the first 24h after the implantation with a peaking high hazard phase in the first postoperative week [31, 32]. The recently published study from Miller *et al*. described possible risk factors for early and late neurogical events occurring in the PARTNER Trial. The risk of late stroke is influenced by patient’s comorbidities [31]. Those factors included advanced functional impairement (NYHA class) and history of stroke within 6 to 12 months before TAVI.

Among all studies, the stroke rate at 2-3 years was different and ranged from 3.9% to 13.5%. Different definitions of neurological events could explain the divergence in the percentage reported in all studies. Such differences in endpoint definitions should be eliminated in the future by using the standardized VARC criteria [33]. 

## CONCLUSIONS

TAVI exhibits excellent hemodynamic and clinical improvement in patients presented with high surgical risk for SAVR. The improvement sustained up to 3 years follow up with a survival rate of approximately 60%. Valve deterioration and tissue endocarditis are very rare even at 3 years follow up. However, the aortic insufficiency after TAVI remains an important issue in this rapidly evolving field.

## Figures and Tables

**Table 1.** Patients characteristics and survival at 30 day, 1-2 and 3-year.

Study	Age (years)	Patients	LES (%)	STS (%)	30-day (%)	1-year (%)	2-year (%)	3-year (%)
Walther *et al*. [18]	82 ± 6	299	31 ± 16	12 ± 8	91	73	68	58
Gurvitch *et al.* [19]	84 ± 7	70	31.7 ± 16	9.6 ± 3.5	n.a	81	74	61
Ussia *et al.* [20]	80.9 ± 6.1	181	24 ± 13.5	11.4 ± 9.9	n.a	76.4	69.7	56.2
Bleiziffer *et al.* [21]	81± 7	227	21 ± 14	7 ± 5	88.5	74.5	64.4	n.a
Buellesfeld *et al*. [14]	81.9 ± 6	126	23.4 ± 13	n.a	84.6	n.a	61.9	n.a

LES indicates logistic EuroSCORE, STS indicates Society of Thoracic Surgeons. n.a: not available.

**Table 2.** The incidence of paravalvular leakage and progression over time.

	Aortic Regurgitation (Moderate or Higher (%))			
	30-day	1-year	2-year	3-year
Walther *et al.* [18]	4	4	5	5
Gurvitch *et al*. [19]	6	4	n.a	n.a
Ussia *et al.* [20]	15.2	17.8	n.a	10
Bleiziffer *et al.* [21]	13	15	14	n.a
Buellesfeld *et al*. [22]	9	3	0	n.a

**Table 3.** Major adverse outcome at 2/3 years.

	Walther *et al*. [18]	Gurvitch *et al*. [19]	Ussia *et al.* [20]	Bleiziffer *et al.* [21]	Buellesfeld *et al.* [22]
Stroke (%)	n.a	8.6	3.9	6.6	13.5
Myocardial infarction (%)	n.a	8.6	1.1	2.7	6.3
Reoperation (%)	n.a	1.4	0	n.a	9.3
Bleeding (major) (%)	n.a	7.1	10.5	4.8	n.a
Endokarditis (%)	n.a	1.4	n.a	n.a	0.8
Structural valve deterioration (%)	n.a	0	0	n.a	0
Valve thrombosis (%)	n.a	0	n.a	n.a	0
